# Neuroplasticity in Human Alcoholism: Studies of Extended Abstinence with Potential Treatment Implications

**DOI:** 10.35946/arcr.v37.1.09

**Published:** 2015

**Authors:** George Fein, Valerie A. Cardenas

**Affiliations:** George Fein, Ph.D., is president and CEO of Neurobehavioral Research, Inc., Honolulu, Hawaii.; Valerie A. Cardenas, Ph.D., is a senior scientist with Neurobehavioral Research, Inc., Honolulu, Hawaii.

**Keywords:** Alcoholism, alcohol use, abuse, and dependence, neuroplasticity, brain, brain networks, appetitive drive networks, appetitive drive, behavior control, inhibitory control networks, functional magnetic resonance imaging, abstinence, electroencephalographic, treatment

## Abstract

Alcoholism is characterized by a lack of control over excessive alcohol consumption despite significant negative consequences. This impulsive and compulsive behavior may be related to functional abnormalities within networks of brain regions responsible for how we make decisions. The abnormalities may result in strengthened networks related to appetitive drive—or the need to fulfill desires—and simultaneously weakened networks that exercise control over behaviors. Studies using functional magnetic resonance imaging (fMRI) in abstinent alcoholics suggest that abstinence is associated with changes in the tone of such networks, decreasing resting tone in appetitive drive networks, and increasing resting tone in inhibitory control networks to support continued abstinence. Identifying electroencephalographic (EEG) measures of resting tone in these networks initially identified using fMRI, and establishing in longitudinal studies that these abstinence-related changes in network tone are progressive would motivate treatment initiatives to facilitate these changes in network tone, thereby supporting successful ongoing abstinence.

A person with alcoholism engages in risky or dangerous drinking despite experiencing serious negative physical and social consequences. Such persistence in pursuing damaging behaviors suggests that the short-term “appetitive” results of drinking (such as intoxication and losing one’s inhibitions) have greater control over the alcoholic’s behavior than do the negative consequences. From a neurobiological perspective, this pattern implies weak “top-down”—or knowledge-driven—executive control over impulsive and compulsive urges to consume alcohol and a strong “bottom-up”—or stimulus-driven—appetitive drive to consume alcohol, both impulsively and compulsively.

Research using functional magnetic resonance imaging (fMRI) has identified networks of disparate brain regions involved in executive control and others involved in appetitive drive. Studies in alcoholics have demonstrated differences in activity in these networks compared with nondrinkers, implying that the networks can contribute to the poor decisionmaking and risky behaviors seen among alcoholics. This article reviews fMRI evidence that, compared with non–substance-abusing control subjects (NSACs), brain executive control networks are weakened or “tuned down” and appetitive drive networks are strengthened or “tuned up” in active alcoholism. Further, alcoholism correlates with changes in synchrony, or how well the brain regions within each network operate in concert. We also present cross-sectional fMRI data showing that abstinence maintenance is associated with compensatory changes in synchrony in these networks, such that the executive control network has greater synchrony and the appetitive drive network has reduced synchrony both in comparison to NSACs. The article proposes that electroencephalographic (EEG) analogs of these alcohol-related network differences exist and should be characterized. EEG could reveal different properties of these brain networks, such as timing of event processing, and may be more amenable than fMRI to active interventions such as neurofeedback. The article reviews a wide literature that supports the potential efficacy of an EEG neurofeedback intervention to mimic or augment the network changes seen in long-term abstinence. Finally, it presents a prototype showing that such neurofeedback is technically feasible.

## Brain Network Activity in Alcoholics

To understand what brain changes underlie behavior seen in alcoholism, researchers have focused on two networks believed to influence whether a person acts to fulfill a desire or to govern or control the desire when faced with a choice. These two networks are the appetitive drive and executive control networks (see sidebar, “Brain Regions and Their Contributions to Behavior”). During its early stages, alcohol consumption is a goal-directed behavior, initiated and executed by regions within the executive control network (such as the dorsolateral pre-frontal cortex and anterior cingulate cortex), with its rewarding effects processed by appetitive drive regions (such as the nucleus accumbens). After a person repeatedly consumes alcohol, consumption may become more automatic (with more involvement of appetitive drive regions such as the caudate and putamen) and less voluntary (with less involvement of executive control regions) (Everitt and Robbins 2005). Alcohol consumption shifts to a more habitual mode, particularly to avoid withdrawal symptoms. The behavioral fate of repetitive actions, such as compulsive alcohol consumption, seem to be instantiated in mesostriatocortical networks (Graybiel 1998; Volkow et al. 2013). An individual with alcohol dependence seeks alcohol compulsively—a behavior associated with increased activity of appetitive drive regions when presented with an alcohol cue—and experiences a lack of engagement of prefrontal regions, which under normal circumstances inhibit or stop a mal-adaptive behavior such as excessive alcohol consumption.

To determine how activity in these brain regions looks among alcoholics compared with control subjects, researchers use fMRI. fMRI measures brain activity by detecting the blood-oxygen-level–dependent (BOLD) contrast related to neural activity. Most fMRI experiments examine task-related patterns in the location and magnitude of the BOLD response, that is, the task activation of the brain. Many differences in activation in the executive control and appetitive drive networks have been observed in alcohol use, abuse, and dependence, suggesting that these networks and the multiple brain regions they encompass can contribute to the poor decisionmaking and risky behaviors seen in alcoholism (for a review, see Camchong et al. 2014).

For example, increased activity in the amygdala and insula, which are associated with inflexible, poor decision-making (Xiao et al. 2013), appears in binge drinkers. Lower activity in the dorsolateral prefrontal cortex (DLPFC) occurs among short-term abstinent alcoholics during inhibition tasks (Li et al. 2009) and in those with a family history of alcoholism during response inhibition (Norman et al. 2011) or when they are asked to make risky versus safe decisions (Cservenka and Nagel 2012). Further, lesser activation of prefrontal executive control regions compared with control subjects has been observed in alcoholics during spatial and verbal working-memory tasks (see the textbox on “Brain Regions and Their Contributions to Behavior”) (Cservenka and Nagel 2012; Desmond et al. 2003; Pfefferbaum et al. 2001). Active drinkers show enhanced BOLD activation in the ventral striatum when presented with visual alcohol cues, which also supports the notion of a stronger appetitive and reward drive in people with current alcohol dependence (Ihssen et al. 2011; Myrick et al. 2004, 2008). Active drinkers with a diagnosis of alcohol dependence compared with active drinkers without alcohol dependence show higher activity in their DLPFCs when performing a delayed-reward decision task (Amlung et al. 2012). This increased activity may reflect increased demand that alcoholics (vs. NSAC) place on the executive control network when required to make decisions to delay behavior ruled by appetitive drive.

These studies demonstrate that excessive alcohol use and even the genetic vulnerability to alcoholism (observed prior to initiating alcohol use) is associated with activation patterns different from those of control subjects in brain regions that are part of the executive control and appetitive drive networks. More recently, scientists have taken fMRI studies a step further to examine differences in how well such brain regions work together. Such work suggests that faulty co-activation or synchrony within brain networks, or an imbalance between opposing brain networks, is important in alcoholism.

## Synchrony in Brain Networks

Various methodologies for detecting brain activity demonstrate that more than one region becomes activated at a time, both during task performance and while at rest. Imaging studies now have begun to parse how the regions work together and whether disturbances within networks are associated with identifiable patterns of behavior. Early fMRI studies primarily focused on changes in the magnitude of the BOLD response, assessing activation and de-activation of brain regions during a task. More recently, studies have shifted to using fMRI to probe the similarity or synchrony of the BOLD response across spatially disparate regions, especially while the brain is at rest. The work builds upon the EEG literature that long ago established the existence of spontaneously oscillating brain networks. EEG measures brain electrical activity. Oscillations detected with EEG at characteristic frequencies, or bands, represent the summed activity of thousands of neurons. Synchrony of oscillatory activity between brain regions is thought to support neural communication and plasticity (for review, see Fell and Axmacher 2011). For example, electrophysiological studies suggest that gamma band (higher than 30 Hz) synchronization is responsible for the integration of brain regions involved in specific aspects of stimulus processing. Synchrony of gamma oscillations enhances neural communication between regions, and lack of synchronization actually may prevent neural communication between cell assemblies. Scientists also have proposed that synchronization facilitates neural plasticity by enabling spike-field coherence that promotes the induction of long-term potentiation in neurons (see Glossary). Supporting this idea, studies show higher phase synchronization during encoding of information that a subject remembers than during encoding of information that the subject does not remember. Thus, scientists typically interpret high correlation or synchrony as representing a more integrated and responsive network and a low correlation or synchrony as representing a dysfunctional network or one with impaired communication. Network synchrony often is referred to in the literature as “functional connectivity.”

Researchers largely agree that cortical oscillations evident in the EEG are related to the BOLD signal detected in fMRI, although the precise relationship is an area of active research (Thompson et al. 2014*a*,*b*; Whitman et al. 2013). This relationship suggests that changes in synchrony of the BOLD response may prove analogous to changes in synchronous EEG oscillations that reflect network integrity. Measuring network synchrony using fMRI can provide more precise information about the locations of brain regions acting together than EEG can capture.

Studies of the synchrony of the fMRI BOLD response during rest have gained in popularity, leading to the identification of several networks that are intrinsic to the brain’s function (for review, see Lee et al. 2013). The most widely studied network is perhaps the default mode network (DMN), which is a group of brain regions that are active at rest but de-activated during cognitive tasks and which exhibits a highly synchronous low frequency (lower than 0.1 Hz) BOLD signal at rest. Many other networks that are highly synchronous at rest have been identified, including the somatosensory, visual, auditory, language, attention, and executive control networks. Networks identified during rest are robust, reliably detected in most people, and remain intact during task performance, although task synchrony may differ from synchrony observed during rest (Wilcox et al. 2011). The success of using synchrony measures in resting state fMRI has led to increased interest in measuring the synchrony of regions of activation in more traditional task-related fMRI studies. This work, in turn, has led to the identification of synchronous networks related to appetitive drive, cue salience, or behavior (Lee et al. 2013), which are key to studies of addiction.

## Resting-State fMRI Synchrony Studies

### Studies in Active Users and Very Early Abstinence

Because synchrony seems to represent the health of a network, it may be affected in certain networks by—or it may affect—alcoholism. Some recent work has examined resting-state fMRI synchrony in multiple brain networks in individuals with current alcohol use disorder (AUD) (Weiland et al. 2014). The fMRI time series measures of synchrony (i.e., average within-network correlations of BOLD signal magnitude across the network’s nodes) were computed for 14 networks in each of 422 individuals with active AUD and in 97 control subjects. In this study, top-down executive control is reflected by the left and right executive control networks (LECN and RECN, respectively). The anterior salience network (composed of nodes including bilateral middle frontal gyrus, middle cingulate gyrus, and insula) reflects bottom-up appetitive drive. Network strength, a global measure of the fMRI time-series synchrony within each network, on average for all networks was lower for subjects with AUD than for control subjects. Tests of single networks showed lower synchrony in subjects with AUD versus control subjects for the LECN, consistent with the model that poor top-down executive control contributes to alcohol dependence. In addition, lower synchrony within the sensorimotor, basal ganglia, and primary visual networks in AUD versus control subjects may reflect alcohol’s damaging effects on other networks that contribute to addiction. For the LECN alone, lower synchrony was associated with greater alcoholism severity and more years of drinking.

A study of fronto-striatal functional connectivity in cocaine use disorders supports the model that a strong bottom-up appetitive drive network is active in addiction (Wilcox et al. 2011). Fourteen subjects with chronic cocaine abuse or dependence (92% with comorbid alcohol abuse or dependence) in very early abstinence (but unlikely to be in significant acute withdrawal) had their resting-state fMRI recorded and compared with that of 16 healthy controls. Patients with chronic cocaine use exhibited increased synchrony between the ventral striatum and orbitofrontal cortex, key regions of the reward and appetitive drive network.

### Studies in Long-Term Abstinence

The above section suggests that current dependence and abuse is associated with exaggerated bottom-up and compromised top-down neural network functioning. The question then becomes whether abstinence from alcohol changes that neural network picture. Existing task studies suggest that compensatory mechanisms appear in long-term abstinence from nicotine and alcohol that may exert control over reward seeking and attenuate appetitive drive (Beck et al. 2009; Grüsser et al. 2004; Nestor et al. 2011; Wrase et al. 2007). To study brain network tone associated with long-term abstinence (LTAA), the authors examined resting-state fMRI synchrony in 23 LTAA subjects (8 women, ages 48.5 ± 7.1 years, abstinent 7.91 ± 7.80 years) and 23 NSAC subjects (8 women, ages 48.0 ± 6.7 years) (Camchong et al. 2013*b*). They used bilateral nucleus accumbens (NAcc) seeds (i.e., the fMRI time-series generated by the left and right NAcc) to probe the reward and appetitive drive network by identifying regions with synchronous fMRI responses, and a subgenual anterior cingulate cortex (sgACC) seed to probe the executive control network. All subjects also performed the intra-/extradimensional set shift task (IED; Cambridge Cognition 2006) outside of the scanner, and the study correlated their performance with the synchrony of the neural networks at rest. The IED assesses cognitive flexibility by examining an individual’s ability to change a learned behavior with changing response contingencies.

**Figure 1:**
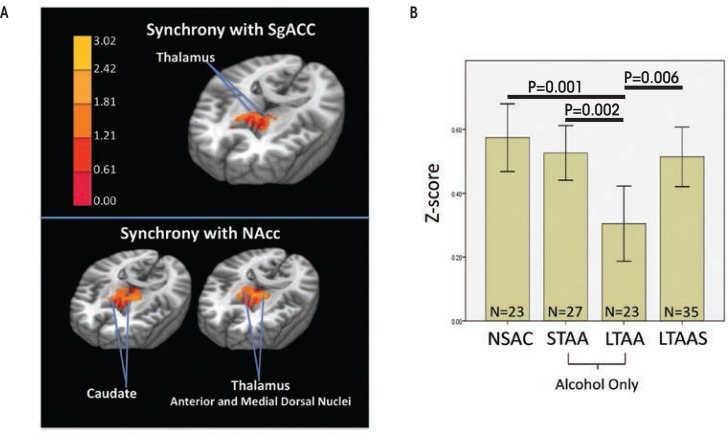
fMRI resting-state synchrony within the appetitive drive network is shown. **(A)** The voxels with activity synchronous to the subgenual anterior cingulate cortex (sgACC) and nucleus accumbens (NAcc) seeds are overlaid in red/yellow. These regions of the thalamus and caudate are crucial in bottom-up appetitive drive. (**B)** The average Z-scores indexing synchrony between the SgACC and NAcc seeds and the colored regions shown in the left panel are shown for non–substance-abusing control subjects (NSAC), short-term abstinent alcoholics (STAA), long-term abstinent alcoholics (LTAA), and stimulus-dependent long-term abstinent alcoholics (LTAAS). The LTAA show significantly less synchrony than NSAC, STAA, and LTAAS, with STAA and LTAAS synchrony midway between NSAC and LTAA.

**Figure 2:**
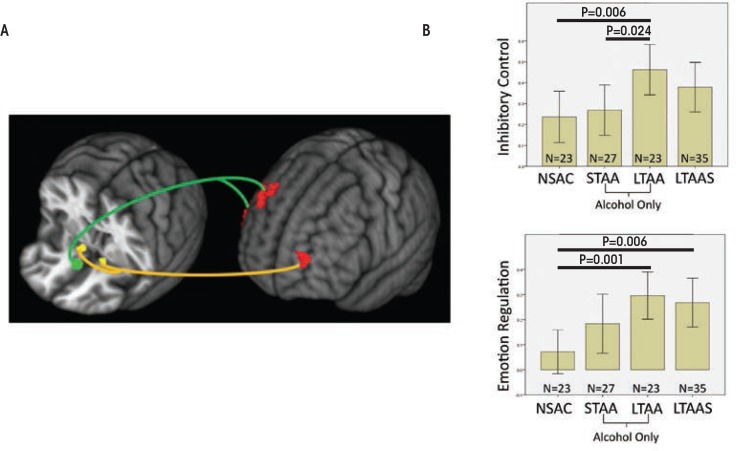
fMRI resting-state synchrony within the executive control network is shown. (**A)** The voxels with activity synchronous with the subgenual anterior cingulate cortex (sgACC, shown in green on the left brain image) are located in the right dorsolateral prefrontal cortex (DLPFC) and are overlaid in red on the right brain image. The voxels with activity synchronous with the bilateral nucleus accumbens (NAcc, shown in yellow) are located in the left DLPFC and are overlaid in red on the right brain image. The right DLPFC is associated with emotion regulation, and the left DLPFC is associated with inhibitory control. **(B)** The average Z-scores indexing synchrony between the NAcc and left DLPFC (top) and between the sgACC and right DLPFC (bottom) are shown for non–substance-abusing control subjects (NSAC), short-term abstinent alcoholics (STAA), long-term abstinent alcoholics (LTAA), and stimulus-dependent long-term abstinent alcoholics (LTAAS). The LTAA show significantly greater synchrony than NSAC and STAA, with STAA and LTAAS synchrony values slightly greater than NSAC, between inhibitory control brain regions. Both LTAA and LTAAS show significantly greater synchrony than NSAC, with STAA values midway between NSAC and LTAA, between emotion regulation brain regions.

Compared with NSAC subjects, LTAA subjects showed (1) decreased synchrony of limbic reward regions (e.g., caudate and thalamus) with both bilateral NAcc and sgACC seeds (figure 1) and (2) increased synchrony of bilateral NAcc seeds with left DLPFC (suggesting greater inhibitory control) and between the sgACC seed and right DLPFC (consistent with greater emotion regulation) (figure 2). The synchrony of bilateral NAcc seeds and left DLPFC was positively correlated with IED task performance outside of the scanner, suggesting that subjects with greater synchrony in the executive control network were better able to inhibit a learned response when a new rule was introduced. Additionally, duration of abstinence in LTAA was negatively correlated with the synchrony between sgACC and right DLPFC.

The lower synchrony of the limbic reward network in LTAA may reflect an ongoing compensatory effort to lower the induction of brain activity in regions known to be involved in reward processing. Increased synchrony between the NAcc and left DLPFC is consistent with literature showing that DLPFC input to the NAcc is involved in inhibition of behavior (Ballard et al. 2011; McClure et al. 2004), as is the correlation of this synchrony measure with IED performance.

LTAA subjects with a shorter duration of abstinence had higher synchrony between sgACC and right DLPFC. The authors suggest that individuals with shorter duration of abstinence are more vulnerable to relapse than individuals with longer abstinence and thus may need more vigilant emotional regulation (reflected here by increased synchrony between sgACC and right DLPFC) to manage emotional situations and successfully avoid relapse. On the other hand, individuals with longer abstinence, who are at lower risk for relapse, may have a lower need for regulating emotion; hence, lower synchrony between sgACC and DLPFC in LTAA subjects was associated with longer (multiyear) abstinence durations. In total, the results here support the existence of compensatory mechanisms in LTAA subjects that are evident during rest, in which enhanced synchrony within the executive control networks and attenuated synchrony within appetitive drive networks may facilitate the behavioral control required to maintain abstinence.

### Studies of Comorbid Stimulant Dependence

To determine whether network synchrony abnormalities also underlie stimulant dependence, we examined LTAA subjects with comorbid stimulant dependence (LTAAS subjects; *n* = 35; 20 women, ages 47.9 ± 7.3 years; averaging 5.67 ± 4.80 years of abstinence), comparing them with 23 LTAA subjects without comorbid drug dependence (Camchong et al. 2013*a*) and 23 NSAC subjects. An earlier finding in this population shows that reduced activity in the insula (see sidebar, “Brain Regions and Their Contributions to Behavior”) in stimulant addicts during decisionmaking (Paulus et al. 2005) or attention tasks (Clark et al. 2012) may predict subsequent relapse. Also, the insula has reciprocal connections with both the executive control (sgACC) and appetitive drive seeds (NAcc) (Craig 2009; Kelly et al. 2012), and accumulating evidence indicates insula involvement in behavioral aspects of addiction such as stress coping, decisionmaking, or cue responsiveness (Naqvi and Bechara 2010). The authors therefore examined synchrony of sgACC and NAcc seeds with insular activity in all three groups. The results showed commonalities in LTAA and LTAAS network synchrony. Compared with NSAC subjects, both groups showed enhanced executive control synchrony and enhanced synchrony between NAcc and midposterior insula. However, differences appeared as well. LTAAS subjects showed no attenuation of their appetitive drive network synchrony, with appetitive drive synchrony presenting higher in LTAAS subjects than LTAA subjects. LTAAS subjects also had enhanced synchrony between sg-ACC and the anterior or mid-insula compared with NSAC subjects. These findings implicate insula involvement in the top-down and bottom-up network adaptive synchrony phenomena in alcohol abstinence, especially in individuals with comorbid drug dependence. These results suggest common as well as specific targets for treatment to support abstinence in chronic alcoholics with, versus without, comorbid stimulant dependence. The results do not speak to possible similar effects in drug addicts without comorbid alcohol dependence, but suggest that studying such individuals with the paradigms presented here may prove fruitful.

### Studies in Short-Term Abstinence

Differences in synchrony observed among abstinent alcoholics compared with control subjects may reflect actual changes that the brain goes through to support abstinence or they may preexist in certain individuals and help those people to achieve and maintain abstinence. If the enhanced executive control network synchrony and suppressed appetitive drive network synchrony observed in LTAA subjects truly represent adaptive network changes during extended abstinence, then similar but smaller magnitude effects on network synchrony should appear in short-term abstinence. The authors investigated whether resting-state fMRI synchrony patterns found in LTAA subjects can be identified in short-term abstinent alcoholics (STAA subjects, abstinent 72.59 ± 18.36 days) (Camchong et al. 2013*c*). Using the same methodology as before (Camchong et al. 2013*b*), they examined network synchrony in 27 STAA subjects, and compared them with the 23 LTAA and 23 NSAC subjects from the previous study. They found synchrony effects ordered in magnitude from NSAC to STAA subjects and then to LTAA subjects within both the appetitive drive and executive control networks. Abstinence duration was associated with progressively lower synchrony of the appetitive drive network (NSAC subjects had higher appetitive synchrony than STAA subjects, who in turn had higher synchrony than LTAA subjects) and higher synchrony of the executive control network (NSAC subjects had lower executive synchrony than STAA subjects, while LTAA subjects demonstrated the highest level of executive control synchrony) (see figures 1 and 2). A significant positive correlation also appeared in STAA subjects between strength of synchrony between NAcc and left DLPFC and IED performance. Finally, the researchers saw a significant positive correlation in STAA subjects between strength of limbic reward network synchrony and current antisocial symptoms (i.e., antisocial behavior). These findings suggest that abstinent alcoholics experience adaptive differences in synchrony patterns compared with control subjects, and the magnitude of the difference increases with duration of abstinence.

## Summary of Resting-State fMRI Synchrony Studies

These studies indicate that active alcoholics exhibit lower top-down executive control network synchrony and higher bottom-up reward and appetitive drive network synchrony, and that these phenomena are more than reversed with successful abstinence. The observed “overcompensation” in network synchrony—that is, the greater executive control network synchrony observed in STAA and LTAA subjects compared with control subjects—may be necessary in order to inhibit the habitual response to alcohol. This is consistent with the authors’ 2013 paper showing that antisocial disposition does not change with long-term abstinence but that antisocial behavior is inhibited, with antisocial symptoms approaching zero in LTAA subjects (Fein and Fein 2013). Given this earlier observation of no change in antisocial disposition (or antisocial thinking) in LTAA subjects, it is not surprising that alcoholics need a very strong inhibitory control system to inhibit antisocial behavior (including drinking).

## Task-Related fMRI Synchrony Studies

Several fMRI task studies have demonstrated altered executive control network activation and connectivity in alcoholism, implying that the resting-state fMRI synchrony differences observed are present during task processing. Research to determine the association between resting state fMRI network synchrony and network performance during tasks that involve appetitive drive and executive control would help demonstrate how the brain’s readiness alters the brain’s response to a task. For example, in nicotine addicts, a modified Stroop task (which tests the time it takes a subject to respond to a question about an image that contains nicotine versus neutral cues) has been used to assess appetitive drive and executive control networks (Nestor et al. 2011). The study provides evidence that higher executive control network activation when viewing nicotine cues occurs in former versus current smokers (i.e., higher executive control network activation appears with longer nicotine abstinence).

**Figure 3:**
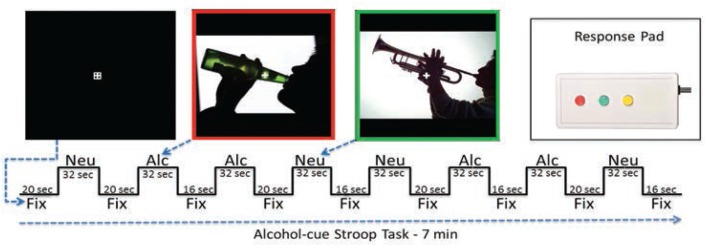
Alcohol-cue Stroop task. During the fixation (Fix) blocks, subjects keep their eyes fixated on the cross. During the neutral (Neu) and alcohol (Alc) blocks, subjects are instructed to keep looking at the fixation cross in the middle, while they notice the color of the picture’s border, and respond by pressing the corresponding colored button on the response pad.

Jazmin Camchong developed an alcohol-cue analog of this task (see figure 3). In a pilot study, she tested five LTAA and two NSAC subjects who had demonstrated resting-state fMRI synchrony differences from each other. She found an alcohol-cue interference effect in LTAA subjects (i.e., longer reaction times to alcohol versus neutral cues) as well as higher synchrony of executive control regions in LTAA versus control subjects when viewing alcohol cues. These pilot results suggest that synchrony within the executive control network is higher in LTAA subjects both at rest and during task performance.

Task studies, which can isolate elements of complex behaviors, could help show not only whether synchrony influences behavior but what synchrony changes mean in relation to what scientists know about how alcoholism disrupts normal functioning. For example, one way of conceptualizing the core problem in alcoholism and other addictions is that reinforcements consequent to behavior—such as becoming sick or hungover after drinking—do not appropriately guide future behavior. Adaptive learning involves computation by the brain of reward prediction errors (PEs), which reflect the difference between expected and actual outcomes. Normally, the PEs affect behavior by influencing higher-order executive functioning of the DLPFC, a region involved in goal-directed behavior. Park and colleagues (2010) tested models of the decision-making deficits in alcoholics and the networks underlying these deficits. They examined striatal PEs and functional connectivity between the striatum and DLPFC. A total of 20 male alcoholics in early abstinence (average 16.9 days abstinent) and 16 male healthy control subjects were studied using fMRI during a reward-guided decision-making task with changing response–outcome contingencies, which assesses how readily the subject learns. Alcoholics needed significantly more trials than did control subjects to meet learning criteria. In both groups, the PE from each stimulus presentation correlated significantly with the BOLD midbrain signal, and there were no differences between groups in the striatal PE signal. However, the influence of the striatal PE signal on the DLPFC was markedly attenuated in the alcoholics, suggesting that although the PE signal was being generated, it did not influence learning in the expected way. Moreover, striatal–DLPFC connectivity correlated significantly with learning during the task and was strongly negatively correlated with craving, especially in alcoholics.

**Brain Regions and Their Contributions to Behavior**

Alcoholic behaviors represent a shift away from regulation of behavior by the brain’s control and management functions (i.e., executive control) and toward influence by functions that process reward (i.e., appetitive drive). Parts of the brain’s complex anatomy involved in each of these functions are spread far apart from one another. Nevertheless, they can act in concert to direct behaviors, and the balance between them turns out to have a profound impact in addiction and recovery. In the human brain, the appetitive drive and reward network—that is, the areas involved in forming and responding to appetites, drives, and desires—comprises mesocortico-limbic regions that mediate aspects of drug addiction such as responses to rewarding stimuli (e.g., the ventral tegmental area and nucleus accumbens), memory of rewarding stimuli (e.g., the amygdala and hippocampus), and regulation of emotion and executive function (e.g., the prefrontal and anterior cingulate cortices) (Everitt and Robbins 2005). The striatum (including the nucleus accumbens, ventral putamen, and ventral caudate) and orbitofrontal cortex are key regions mediating appetitive drive and behavior toward seeking reward (Elliott et al. 2010; Everitt and Robbins 2005; Taha and Fields 2006).

The subgenual anterior cingulate cortex (sgACC), a subdivision of the anterior cingulate cortex, plays a central role within the predominantly frontal cortical network underlying executive control (Botvinick et al. 2001). The ACC has widespread connections with the lateral prefrontal cortex and limbic structures (including the hippocampus, amygdala, and anterior thalamus) that are involved in emotional responsiveness and the regulation of behavior in the context of rewarding and punishing outcomes (Drevets et al. 1997; Kelly et al. 2009; Phan et al. 2005). A compromised top-down executive control network may underlie the poor regulation of behavior and emotion that has been considered primary in relapse (Berking et al. 2011; Cooper et al. 1995; Fox et al. 2008).

Here, images of the brain are labeled with some of the regions most important to the executive control and appetitive drive networks. The behaviors with which the regions are associated are also listed.

**Appetitive Drive Network**

**Amygdala:**

*See* limbic system.

**Caudate:**

Part of the striatum that influences goal-directed actions or behaviors.

**Hippocampus:**

*See* limbic system.

**Insula:**

Implicated in inflexible, poor decisionmaking. Also involved in stress coping and cue responsivity, which are behavioral aspects of addiction.

**Nucleus Accumbens:**

Part of the striatum with roles in reward and reinforcement learning as well as fear, impulsivity, and addiction.

**Orbitofrontal Cortex:**

Involved in motivational behavior as well as emotion and social behavior. It receives and responds to primary sensory information. It is involved in the detection and processing of consequences of behavior, including the attachment of emotional valence to the negative consequences of behavior.

**Posterior Cingulate Cortex:**

Part of the default mode network (see Glossary) and possibly involved in human awareness. Also involved in pain and episodic memory retrieval and in intrinsic control networks.

**Prefrontal Cortex:**

Involved in planning cognitive behavior, regulation of emotion, and executive function. Also part of the executive control network.

**Putamen/Ventral Putamen:**

Part of the striatum involved in mediating appetitive drive. The putamen regulates movement and has influence on habits and on learning related to stimulus response.

**Thalamus:**

*See* limbic system.

**Ventral Tegmental Area:**

Involved in response to rewarding stimuli.

**Executive Control Network**

**Basal Ganglia:**

Connected with the cerebral cortex, thalamus, and brain stem. Involved in the control of voluntary movement, procedural learning, habits, cognition, and emotion.

**Bilateral Middle Frontal Gyrus/Middle Cingulate Gyrus:**

Parts of a salience network, a key mechanism by which the brain picks out details in its environment to focus on. Involved in learning and attention.

**Lateral Prefrontal Cortex:**

Involved in goal-directed behavior. Includes the dorsolateral prefrontal cortex (a functional distinction), involved in executive functions such as working memory, cognitive flexibility, planning, inhibition, and abstract reasoning.

**Limbic System:**

Encompasses the hippocampus, amygdala, and anterior thalamus. Implicated in both appetitive drive and executive control networks. An emotion, behavior, and motivation center.

**Figure:**
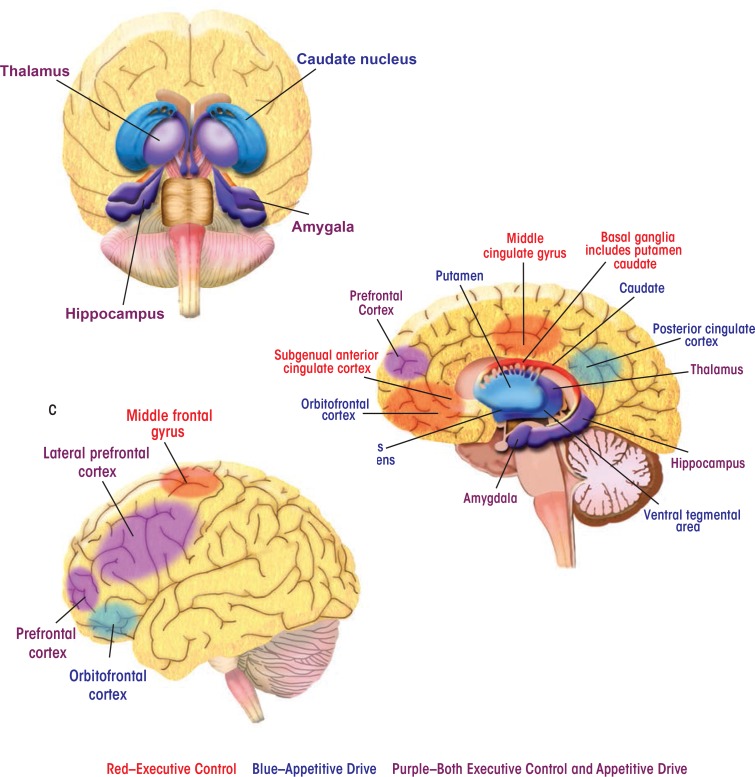
Locations of brain regions involved in executive control and appetitive drive. **(A)** Front Brain View: A frontal image of the brain showing internal structures involved in appetitive drive and in both appetitive drive and executive control networks. Though spread far apart in the brain’s anatomy, the regions (shown here and in the other two brain illustrations) operate in concert to form these networks. **(B)** Side Brain View: A side view of the brain showing internal structures and locations of regions associated with either executive control or appetitive drive or, in many cases, with both networks. **(C)** External Brain View: An external view of the brain showing regions associated with the appetitive drive and executive control networks.

**Subgenual Anterior Cingulate Cortex (sgACC):**

Connected with the lateral prefrontal cortex and with limbic system regions. Involved with emotion processing, learning, and memory.

ReferencesBerkingMMargrafMEbertDDeficits in emotion-regulation skills predict alcohol use during and after cognitive-behavioral therapy for alcohol dependenceJournal of Consulting and Clinical Psychology79330731820112153465310.1037/a0023421PMC3109184BotvinickMMBraverTSBarchDMConflict monitoring and cognitive controlPsychology Review108362465220011148838010.1037/0033-295x.108.3.624CooperMLFroneMRRussellMDrinking to regulate positive and negative emotions: A motivational model of alcohol useJournal of Personality and Social Psychology6959901995747304310.1037//0022-3514.69.5.990DrevetsWCPriceJLSimpsonJRSubgenual prefrontal cortex abnormalities in mood disordersNature38666278248271997912673910.1038/386824a0ElliottRAgnewZDeakinJFHedonic and informational functions of the human orbitofrontal cortexCerebral Cortex20119820420101943570710.1093/cercor/bhp092EverittBJRobbinsTWNeural systems of reinforcement for drug addiction: From actions to habits to compulsionNature Neuroscience8111481148920051625199110.1038/nn1579FoxHCHongKASinhaRDifficulties in emotion regulation and impulse control in recently abstinent alcoholics compared with social drinkersAlcoholism: Clinical and Experimental Research33238839420081802329510.1016/j.addbeh.2007.10.002KellyAMDi MartinoAUddinLQDevelopment of anterior cingulate functional connectivity from late childhood to early adulthoodCerebral Cortex19364065720091865366710.1093/cercor/bhn117PhanKLFitzgeraldDANathanPJNeural substrates for voluntary suppression of negative affect: A functional magnetic resonance imaging studyBiological Psychiatry57321021920051569152110.1016/j.biopsych.2004.10.030TahaSAFieldsHLInhibitions of nucleus accumbens neurons encode a gating signal for reward-directed behaviorJournal of Neuroscience26121722220061639969010.1523/JNEUROSCI.3227-05.2006PMC6674301

In another study, 20 non–treatment-seeking problem drinkers underwent fMRI during a stop-signal task (SST) to assess response inhibition, a subject’s ability to inhibit his own response to a stimulus (Courtney et al. 2013). Weaker functional connectivity between frontal regions and the striatum correlated with the severity of alcohol dependence, although SST behavioral performance was uncorrelated with severity, suggesting that the BOLD signal is more sensitive to alcohol’s effects than task performance. The researchers concluded that as alcoholism progresses, the fronto-striatal pathway is weakened, leading to less inhibitory control as part of executive functioning.

Other studies of network functioning in alcoholics during active tasks have also revealed abnormalities in networks other than the executive control and appetitive drive networks. For some tasks, alcoholics seemed to recruit additional brain regions (vs. controls) to accomplish a task, perhaps to overcome strong appetitive signals, or physical or functional degradation of brain networks used by controls for task performance. Within the DMN, for example, abstinent alcoholics show less resting-state synchrony between the posterior cingulate and cerebellar regions compared with control subjects but show greater left posterior cingulate-cerebellar synchrony during a spatial working-memory task. The finding suggests that alcoholics need more integration of inputs from multiple brain regions to achieve comparable task performance to controls (Chanraud et al. 2011). In addition, higher connectivity among nodes of the DMN was associated with better task performance in both alcoholics and control subjects and also associated with longer abstinence in the alcoholics.

In later work (Chanraud et al. 2013), researchers observed that compared with control subjects, recovering alcoholics recruited two additional fronto-cerebellar networks during a spatial working-memory task. In another study, lower fronto-cerebellar fMRI synchrony during a motor task also was observed in chronic alcoholics who were abstinent 5 to 7 days versus control subjects (Rogers et al. 2012). These results reinforce the idea that people generally require synchronous brain activity from disparate regions to respond appropriately to a stimulus and that alcoholics may need to marshal more brain regions to complete a task. The finding also provides evidence for improved network communication with extended sobriety.

A study of 18 abstinent alcoholics and 17 healthy control subjects acquired fMRI data during an attentional Stroop task (Schulte et al. 2012) and revealed abnormal synchrony in networks in the brains of abstinent subjects that may mediate between the top-down executive control and bottom-up appetitive drive networks. Using midbrain or posterior cingulate cortex (PCC) seeds (regions showing significant group-by-task activation contrasts in the fMRI analysis), the authors observed lower synchrony in alcoholics versus control subjects between the PCC and middle cingulate cortex, which they interpreted as reflecting difficulty in adapting functional network activity to executive task demands. They also observed greater synchrony between the midbrain and the middle cingulate cortex and striatal regions. They believe this suggests that alcoholics rely on greater integration of inputs from multiple brain regions as a compensatory mechanism to support task performance.

Task-related fMRI studies also may help identify characteristics of brain connectivity that can help predict whether or how readily an alcoholic will achieve abstinence. A cue-reactivity fMRI experiment with alcohol-associated and neutral stimuli was used to study 46 detoxified alcohol-dependent patients (19.74 ± 22.66 days abstinent) and 46 control subjects (Beck et al. 2012). Three months following scanning, 30 patients had relapsed and 16 had maintained alcohol abstinence. The study compared fMRI results of the subsequent relapsers with those of the abstainers. When presented with alcohol-associated versus neutral stimuli, abstainers had demonstrated stronger functional connectivity than those who had relapsed between mid-brain (including the ventral tegmental area and subthalamic nuclei) and left amygdala and between midbrain and left orbitofrontal cortex. These are brain regions associated with the processing of salient or aversive stimuli. The increased synchrony in abstainers between the midbrain and amygdala may mediate an enhanced aversive reaction to alcohol stimuli, which may then act as a warning signal (through stronger midbrain-frontal cortex synchrony) to help maintain abstinence.

In summary, fMRI functional connectivity or synchrony studies provide ample evidence that altered network synchrony exists in alcoholism and that plastic changes in network synchrony occur with abstinence. However, from cross-sectional studies alone, one cannot distinguish between brain synchrony actually changing in long-term abstinence (Camchong et al. 2013*b*,*c*), versus selective survivorship (i.e., individuals with such synchrony differences are more likely to achieve abstinence, and individuals with the largest differences from NSAC are more likely to achieve protracted abstinence), or a combination of the two. Only longitudinal studies can determine whether the observed cross-sectional findings indeed reflect adaptive changes in network synchrony with extended abstinence.

## Applying Synchrony Findings to Treatment

Scientists understand little of how successful treatments such as behavioral therapies or 12-step programs work. They also understand little of the neurological mechanisms underlying reduction or cessation of drinking. Data reviewed here point to one such possible mechanism. They reveal network synchrony changes detected using fMRI that are graded with abstinence duration, suggesting that achieving and maintaining abstinence is associated with adaptive brain network synchrony changes that support reductions in bottom-up appetitive drive and increases in top-down executive inhibitory control. If longitudinal studies can confirm that the degree of the changes in the appetitive drive and executive control networks is associated with and predictive of successful abstinence, then such changes may underlie the success of behavior therapies. In addition, interventions that directly augment the network changes may provide another tool in the treatment toolbox.

The idea of modifying brain network synchrony to promote abstinence is bolstered by the literature on using transcranial direct-current stimulation (tDCS) or repetitive transcranial magnetic stimulation (rTMS) to treat alcohol craving. These noninvasive treatments are thought to reduce craving by modulating the activity and connectivity of brain networks. Boggio and colleagues (2008) showed that tDCS of the DLPFC decreased alcohol craving compared with sham treatment. In later work, Mishra and colleagues (2010) studied 45 alcohol-dependent patients administered rTMS of the DLPFC and found significant decreases in a craving measure within the group that received rTMS compared with the sham group. One interpretation is that these treatments resulted in increased DLPFC activity and better executive control over craving.

A case study by De Ridder and colleagues (2011) provides further evidence that brain functions in alcoholism can be trained or influenced using relatively noninvasive techniques. The researchers used rTMS targeting the anterior cingulate cortex in an attempt to reduce craving and promote abstinence in a woman with a long history of alcohol dependence and treatment. Before treatment, the patient showed increased EEG synchrony between the ACC and PCC, and fMRI showed activation of regions of the appetitive drive network (NAcc, ACC, and PCC) in response to cue-induced worsening of craving. Following successful rTMS, fMRI-detected activation of NAcc, ACC, and PCC disappeared, and the patient’s synchrony pattern normalized. When rTMS treatment became ineffective and relapse occurred, activity and synchrony within the appetitive drive network returned. Although their effect was not permanent, the rTMS treatments seem to have altered network synchrony and reduced craving.

Direct currents and magnetic waves applied transcranially thus seem to influence brain synchrony and may help reduce symptoms such as craving in alcoholism. At the same time, people can achieve abstinence without them. A technique such as neurofeedback might help people with addictions directly strengthen the tone of their inhibitory networks or weaken the tone of their appetitive drive networks. Neurofeedback is a method built upon the idea that the mind and body are one, and that by training the mind or brain to achieve particular states indexed by some measured neurobiological signal (such as the BOLD response or EEG), the body will react in a more optimal way in order to improve emotional, cognitive, physical, and behavioral experiences. Neurofeedback that “feeds back” an auditory or visual signal that corresponds to the strength of brain network synchrony may promote network synchrony adaptations that support abstinence. For example, a neurofeedback protocol may instruct a patient to try to raise the pitch of a tone. A low-pitched tone is played when network synchrony is low, and the pitch increases with network synchrony. As the patient works to raise the tone, synchrony in the target network improves, training the network.

### Relating fMRI to EEG

Some technical challenges stand in the way of neurofeedback. First, it is neither practical nor economically feasible to use neurofeedback to modify fMRI-detected network synchrony directly. Furthermore, fMRI’s BOLD response cannot provide the time resolution necessary to allow real-time feedback to a patient about synchrony changes occurring in his or her brain. In contrast, EEG provides precise time resolution and generally is a more economical and efficient tool for use in treatment than fMRI.

Since research on brain networks involved in alcoholism has used fMRI to date, scientists need to find EEG results that are analogous to the relevant fMRI-detected network phenomena to make EEG useful in neurofeedback. Fortunately, converging evidence suggests that the fMRI BOLD response reflects the summed neural activity of several oscillatory EEG networks (for review, see Whitman et al. 2013). These EEG networks may oscillate out of phase (i.e., the peak of oscillation does not coincide across nodes of the network) at multiple frequencies (e.g., theta, alpha, or gamma), and the activity of separate networks may vary as a function of cognitive states lasting only a few hundred milliseconds. fMRI networks detected in response to task processing are likely to comprise multiple oscillatory EEG networks reflecting both evoked (i.e., time-locked to the task) and induced (i.e., not time-locked) EEG responses and including responses that derive from phase alignment within EEG networks, wherein the summed activity creates a large, detectable signal (Burgess 2012). Because of the more complex nature of EEG measures of brain activity that change at the same pace as cognitive processes, EEG networks representing executive control and appetitive drive could potentially reveal more about the mechanisms underlying the processing and inhibition of alcohol cues that contribute to the maintenance of abstinence. Such EEG networks also could serve as neurofeedback targets.

## Neurofeedback of EEG Network Synchrony

EEG network connectivity analysis is in its early stages, but pursuit of the identification of EEG networks that change with abstinence is crucial given the possibility of a neurofeedback intervention to facilitate abstinence. Preliminary data show that resting EEG coherency carries information that differs between LTAA and NSAC subjects, and that correlates with resting-state fMRI executive control network synchrony. Further study could identify reliable EEG executive control and appetitive drive network synchrony measures as neurofeedback targets.

Roberto Pascual-Marqui’s keynote address at the International Society for Neurofeedback and Research in 2011 presented a model for examining brain network synchrony from scalp-recorded EEGs. Using low-resolution electromagnetic tomography (LORETA) (Pascual-Marqui 2002, 2007) to estimate cortical EEG sources and independent components analysis (ICA) to identify synchronous source activity, he demonstrated EEG networks involving similar cortical regions to those identified by resting state fMRI from the literature. More recent work used EEG to study the effect of acute alcohol intake on the brain’s resting state network in social drinkers. It examined the coherence between the activity of certain cortical areas within different frequency bands (Lithari et al. 2012) to construct brain networks. The work demonstrates that network synchrony changes occur over a short period of time (within 25 minutes of alcohol consumption) and are reflected in the scalp-recorded EEG, which can then be attributed to brain locations for network analysis. These results support the idea that EEG brain network synchrony could provide a neurofeedback target.

### History

EEG neurofeedback in the treatment of substance use disorders dates to 1975 (for review, see Sokhadze et al. 2008) and was based on an alpha-theta training protocol, aimed at increasing the proportion of alpha (8 to 13 Hz) and theta (4 to 7 Hz) band activity in the ongoing EEG to promote a state of profound relaxation similar to a meditative state. Although early studies were uncontrolled and abstinence rates were not reported, results suggested that biofeedback-induced alpha/theta states promoted insight and attitude changes in alcoholics, and that these changes enhanced recovery (Twemlow and Bowen 1976, 1977; Twemlow et al. 1977). Peniston and Kulkosky (1989) conducted the first randomized controlled studies of alpha-theta EEG neurofeedback. Of 10 alcoholic patients (who had formerly failed hospital treatment for alcoholism) who underwent neurofeedback training, 8 remained generally abstinent for at least 3 years, and they showed persistent changes in alcoholic personality variables. A case study (Fahrion et al. 1992) further described neurofeedback treatment in an 18-month-abstinent alcoholic who was experiencing craving and a fear of relapse. It concluded that neurofeedback was a useful intervention for reducing craving even in abstinent alcoholics. Later work also reported sustained abstinence in a group of alcoholic depressed patients who were treated with alpha-theta neurofeedback (Saxby and Peniston 1995). Critics deem alpha-theta neurofeedback no more effective than suggestion or meditation techniques. However, the fact that feedback of a single electrode measuring alpha and theta—which affords a limited view of the complex interaction of brain networks involved in alcohol abuse and dependence—works as well as it does, encourages the notion that feedback of EEG signals reflecting the functioning of the executive control and appetitive drive networks would yield even more impressive results.

To examine this idea that neurofeedback learning would be improved if activity from specific brain regions related to the desired outcome behavior was monitored, Congedo and colleagues (2004) pioneered neurofeedback using LORETA with a protocol designed to improve sustained attention. Alpha and beta band current densities were estimated for an anterior cingulate region of interest using LORETA based on 19 scalp electrodes, and the power ratio between bands was used to drive feedback signals. They demonstrated that the current density power ratio increased over multiple neurofeedback sessions and that subjects could willfully increase that ratio. Scientists subsequently used LORETA neurofeedback to train eight healthy individuals to increase their low-beta power activity (moving the EEG frequencies in a direction opposite to alpha/theta feedback) for an anterior cingulate ROI in an effort to improve alertness and attention (Cannon et al. 2007). The subjects increased their beta power within the target ROI after neurofeedback, and these changes were associated with behavior change. Furthermore, beta power increases also were observed within ROIs that encompassed the left and right prefrontal cortex and the right post central gyrus, demonstrating parallel modifications in regions of the executive control network, although training targeted only a single anatomical node. More recent work has explored the feasibility of neurofeedback using a LORETA-derived anatomical source in clinical populations (Cannon et al. 2008) and has explored the utility of measuring EEG network synchrony using LORETA-derived sources (Cannon et al. 2012; Coben et al. 2014).

### EEG Neurofeedback

The authors propose that EEG neurofeedback promoting increased inhibitory control network synchrony and reduced appetitive drive network synchrony would result in a “resting-state brain” that can more appropriately deal with the challenges of maintaining abstinence. The design of such an EEG neurofeedback protocol requires identification of EEG networks that change with abstinence and correspond to the appetitive drive and executive control networks previously identified using fMRI. Given the success of LORETA for estimating EEG network synchrony (Cannon et al. 2012; Coben et al. 2014; De Ridder et al. 2011) and the active research in the estimation of EEG sources and source synchrony (Chiang et al. 2009; Cook and Koles 2006; Gramfort et al. 2013; Sekihara et al. 2001), these networks likely can be identified and used as neurofeedback treatment targets for abstinence maintenance.

**Cognitive Testing Tools**

Alcoholism affects an array of cognitive functions that involve different brain regions. Asking a patient or subject to perform tasks that isolate specific cognitive processes from each other provides an essential tool for imaging studies, because the tasks induce measurable activity in the specific brain regions required to perform them. Researchers can compare activity patterns seen among alcoholics with those seen among abstainers and healthy control subjects. Tests referred to in this article are described here:

**Delayed Reward Task:**

This tests a subject’s ability to resist the temptation of an immediate reward in favor of waiting for a later reward. The task involves impulse control and self-control.

**Intra/Extradimensional Set Shift Task:**

This tests the subject’s ability to learn a rule through trial and error and then reverse it in favor of a new rule. The task requires attention and flexible thinking.

**Motor Task:**

The task tests a subject’s ability to learn and voluntarily produce intentional movements to proficiently perform a goal-oriented task. Motor tasks require considerable cognitive input.

**fMRI Reward-Guided Decision-Making Task:**

This assesses a subject’s learning rate by letting the subject look at different stimuli and choose one that is associated with a positive outcome (e.g., a smiley face). Each time the subject chooses an item and receives negative feedback (e.g., a frowning face), a prediction error is generated. The learning rate counts the number of trials the subject goes through to figure out which stimulus leads to a positive outcome.

**Spatial and Verbal Working-Memory Tasks:**

Working memory actively holds multiple pieces of information in the mind where they can be manipulated. It includes subsystems that store and manipulate both visual images and verbal information. Tasks that test working memory require a subject to manipulate information as part of a goal-directed action while also being presented with distractions. The cognitive processes required to accomplish the task include executive control and attention, among others.

**Stop-Signal Task:**

Here, a subject is asked to respond as quickly as possible to a particular feature of a stimulus (e.g., color, shape, or location). In some instances, however, the stimulus is followed by another signal—such as an auditory tone—that tells the subject to withhold her planned response. This tests the subject’s ability to inhibit responses.

**Stroop Task:**

This assesses whether a subject experiences interference in reaction time for completing a task. The classic Stroop test example involves looking at the names of colors spelled out in ink that is not the same color as the word (e.g., the word “red” spelled in blue ink). The subject is asked to name the color of the ink, and reaction time can indicate whether a person has problems with selective attention, cognitive flexibility, or processing speed.

Technical challenges are inherent in a real-time EEG brain network synchrony neurofeedback system. However, the authors’ prototype for an EEG neurofeedback system uses a quad-core Intel i5 computer to acquire EEG and estimate network synchrony based on comparing the EEG of each possible pair of electrodes, and a second computer to display a movie as the feedback signal. Although the best estimates of EEG network synchrony likely will be derived from intracranial source estimates, the prototype has computational demands greater than those required to estimate intracranial source connectivity and thus is more than adequate to establish the feasibility of a future EEG network synchrony neurofeedback system. The prototype records 64 channels of scalp EEG, estimates pairwise cross-coherencies, and computes the contribution of the independent components (IC) that index executive control or appetitive drive network synchrony. First, the subject’s baseline network synchrony is estimated for use during training. During neurofeedback training, the system continuously records 64 channels of scalp EEG and analyzes the EEG to estimate network synchrony in real time. The real-time synchrony is compared with the subject’s baseline synchrony and the target distributions of synchrony for NSAC, STAA, and LTAA subjects. A degraded video stimulus feeds back to the subject if there is a large difference between the real-time estimate of synchrony and the target synchrony, whereas a clear video signal appears when the real-time synchrony estimate approaches the target synchrony. The prototype is fast enough to update the neurofeedback to the patient 10 times per second despite a computationally intensive method of reflecting EEG network synchrony. It is likely that a much simpler algorithm will sufficiently index EEG network synchrony once research clarifies which signals best represent key aspects of brain network synchrony in recovering alcoholics. For example, neurofeedback systems could eventually use cross-correlation of selected electrode pairs within one or two frequency bands, or correlation of estimated source activity or power between a small number of anatomical sources. The central research task that would enable development of an EEG neurofeedback system to treat alcoholism remains identifying the EEG measures of network function that change with abstinence and that correspond to the appetitive drive and inhibitory control fMRI networks.

## Conclusions

Alcoholism is characterized by a lack of control over excessive alcohol consumption despite significant negative consequences, a pattern of behavior that implies weak top-down executive control over impulsive and compulsive urges to consume alcohol, and a strong bottom-up appetitive drive that produces those urges. fMRI studies have identified multiple brain regions that contribute to the poor decisionmaking and risky behaviors seen in alcoholism. This chapter reviews fMRI network synchrony, or functional connectivity, studies suggesting that faulty coactivation or synchrony of multiple brain regions comprising networks, or an imbalance between opposing brain networks, is important in alcoholism. fMRI network studies in active alcoholics suggest that impulsive and compulsive behaviors are related to the ineffectiveness of brain networks, characterized by decreased synchrony in top-down executive control network and increased synchrony in the bottom-up appetitive drive network. Repeated high-volume alcohol exposure may compromise network integrity, as suggested by the relationship between synchrony and the severity and duration of alcohol use. Continued abstinence following alcoholism displays a different synchrony pattern. A series of studies in short- and long-term abstinent alcoholics observed decreased synchrony in appetitive drive networks and increased synchrony in inhibitory control networks, suggesting that the alcohol-induced imbalances in brain networks are reversed, helping individuals achieve and maintain abstinence by inhibiting behavior and reducing appetitive drive. Longitudinal studies of abstinent alcoholics at rest and during task performance would definitively establish whether plastic changes in the synchronous activity in brain networks reflects a crucial brain mechanism underlying the behavior changes in alcoholics that result in extended abstinence. Furthermore, the identification of EEG measures analogous to fMRI-executive control and appetitive drive network synchrony could potentially reveal the sequence and timing of mechanisms underlying the processing and inhibition of the brain’s response to alcohol cues that contribute to the maintenance of abstinence. Confirming the progressive network synchrony changes with longitudinal studies of abstinent alcoholics—together with identifying EEG networks—would support the treatment potential of interventions to augment these network changes. Neurofeedback of EEG alpha and theta rhythms has been a successful component of alcoholism treatment in some subjects, and feedback of a signal that indexes synchrony in specific brain networks holds great promise as an alcoholism treatment. A prototype for neurofeedback to alter measures of EEG network synchrony demonstrates the technical feasibility of this treatment approach. If longitudinal studies confirm that the adaptive changes in brain functional organization summarized in this article support ongoing abstinence, then EEG treatment to augment these changes is feasible and should be pursued.

## Notes

Glossary

Default mode network (DMN): A network of defined brain regions that is active when a person is not focused on the outside world but is awake. It is characterized by neural oscillations (see electroencephalography [EEG], below) and is deactivated when a person focuses on a task or action.
Electroencephalography (EEG): Records electrical activity along the scalp. EEG measures voltage fluctuations resulting from activity in the neurons of the brain. It can detect neural oscillations, which reflect the naturally occurring rhythmic, repetitive neural activity that occurs in the central nervous system. When many neurons act together, the synchronized activity results in the oscillations. Different synchronized activity between neurons gives off oscillations at different, characteristic frequencies. These frequencies have been aggregated into bands that have been named with Greek letters (e.g., alpha, theta, gamma, etc.).
Functional Magnetic Resonance Imaging (fMRI): A technique for assessing brain activity by measuring changes in blood flow that occur in response to neural activity (also called the BOLD response). MRI uses an electromagnet to align atomic nuclei, which then give off a measurable signal. MRI measures the magnetic signal from hydrogen nuclei in water. When neurons increase their activity, their demand for oxygen increases and blood flow increases to the area, allowing the system to determine what brain regions are active versus others.
fMRI Seeds: Signals from very precise locations in the brain region or structure of interest. Seeds can be a single fMRI volume element or a “region of interest” or ROI. Seeds are used to calculate correlations with the activity of all other locations, which appear as connections “growing” from the “seed,” resulting in detailed data on connectivity in brain areas.
fMRI Task-Related Studies: Record and measure activation of brain regions while a subject is asked to complete a task, such as looking at a picture, that elicits a specific cognitive response in the brain. Studies can be designed so that the process of interest can be measured separately from other processes (see textbox).
Low-Resolution Electromagnetic Tomography (LORETA): A method for determining the location of electrical activity in the brain using multiple channel electroencephalography recordings.
Neurofeedback: Biofeedback that uses real-time displays that are a function of brain activity—including electroencephalography—to teach self-regulation of brain function.
Phase Synchronization: Occurs when a certain characteristic of an oscillation—the phase—is aligned in separate brain regions. When oscillations are in phase or synchronized, they reinforce each other.
Repetitive Transcranial Magnetic Stimulation (rTMS): Uses precisely targeted magnetic pulses to stimulate areas of the brain.
Spike-Field Coherence: Measures neuronal synchronization across brain areas by comparing spikes—electrical signals that occur when a neuron “fires” or emits an action potential—with the surrounding field potential, which is the compound activity of a large pool of neurons that may oscillate at different frequencies. Spikes may be synchronized or “coherent” at some frequencies that contribute to the local field potential, but may have no phase relation to other frequencies.
Synchrony: Oscillatory activity of physically distant brain regions occurring at the same time (or coinciding). Synchronization has been linked to cognitive functions.
Transcranial Direct Stimulation (tDCS): Uses constant, low current to stimulate a brain region, delivered to the brain region through scalp electrodes.

## References

[b1-arcr-37-1-125] Berking M, Margraf M, Ebert D (2011). Deficits in emotion-regulation skills predict alcohol use during and after cognitive-behavioral therapy for alcohol dependence. Journal of Consulting and Clinical Psychology.

[b2-arcr-37-1-125] Botvinick MM, Braver TS, Barch DM (2001). Conflict monitoring and cognitive control. Psychology Review.

[b3-arcr-37-1-125] Cooper ML, Frone MR, Russell M (1995). Drinking to regulate positive and negative emotions: A motivational model of alcohol use. Journal of Personality and Social Psychology.

[b4-arcr-37-1-125] Drevets WC, Price JL, Simpson JR (1997). Subgenual prefrontal cortex abnormalities in mood disorders. Nature.

[b5-arcr-37-1-125] Elliott R, Agnew Z, Deakin JF (2010). Hedonic and informational functions of the human orbitofrontal cortex. Cerebral Cortex.

[b6-arcr-37-1-125] Everitt BJ, Robbins TW (2005). Neural systems of reinforcement for drug addiction: From actions to habits to compulsion. Nature Neuroscience.

[b7-arcr-37-1-125] Fox HC, Hong KA, Sinha R (2008). Difficulties in emotion regulation and impulse control in recently abstinent alcoholics compared with social drinkers. Alcoholism: Clinical and Experimental Research.

[b8-arcr-37-1-125] Kelly AM, Di Martino A, Uddin LQ (2009). Development of anterior cingulate functional connectivity from late childhood to early adulthood. Cerebral Cortex.

[b9-arcr-37-1-125] Phan KL, Fitzgerald DA, Nathan PJ (2005). Neural substrates for voluntary suppression of negative affect: A functional magnetic resonance imaging study. Biological Psychiatry.

[b10-arcr-37-1-125] Taha SA, Fields HL (2006). Inhibitions of nucleus accumbens neurons encode a gating signal for reward-directed behavior. Journal of Neuroscience.

